# Copper Sulfide Nanoparticles Anchored in Cotton Linter Carbon Aerogel Promote the Adsorption/Photocatalytic Degradation of Organic Pollutants

**DOI:** 10.3390/gels11110931

**Published:** 2025-11-20

**Authors:** Yueyuan Xu, Yuxuan Guo, Canming Hu, Yueqi Zhou, Chengli Ding

**Affiliations:** National Key Laboratory for Carbon-Based Energy Resource Chemistry and Utilization, Jointly Constructed by the Province and the Ministry, Xinjiang University, Urumqi 830046, China; 13899507690@163.com (Y.X.); gyx010323@163.com (Y.G.); m17690288062@163.com (C.H.); zhouyueqiqi@126.com (Y.Z.)

**Keywords:** copper sulfide, short-staple cotton, photodegradation, carbon aerogel, printing and dyeing wastewater

## Abstract

The development of cheap and efficient photocatalysts for the degradation of organic pollutants in textile printing and dyeing wastewater is of great importance for addressing environmental issues, although it remains challenging. In this study, nano-CuS particles were doped on cotton linter aerogels using a straightforward method for the degradation of methylene blue (MB) and organic pollutants in textile wastewater. Material morphology and structure were analyzed using XRD, SEM/EDS mapping, XPS, BET surface area measurements, and UV-Vis spectroscopy, while their performance was evaluated through various tests. The results demonstrated that a 10 mg catalyst material achieved complete degradation of a 20 mL methylene blue solution (15 mg/L) within 120 min. Moreover, the degradation rates of two types of textile wastewater, reactive red wastewater and reactive yellow wastewater, were both above 90% within 120 min and reached complete degradation within 150 min using the 10 mg catalyst material. The experimental results demonstrate that copper sulfide nanoparticles anchored in cotton linter carbon aerogel can increase the contact area of the photocatalytic reaction system, improve the photoelectron transfer, and thus enhance the photocatalytic reaction efficiency, providing a useful foundation for developing economical photocatalysts and effective dye degradation technologies.

## 1. Introduction

Wastewater from textile printing and dyeing is typically characterized by high pH, high salinity, intense color, and complex composition, making it one of the most challenging industrial wastewaters to treat, and the urgency of addressing wastewater treatment has been escalating [1,2]. Industrial dyes such as methylene blue pose significant risks to both ecosystems and human health. Their discharge contributes to water eutrophication, inhibits aquatic organism photosynthesis, and presents carcinogenic and allergenic threats through ingestion or skin contact [3]. The inherent chemical stability of these dye molecules results in residual rates exceeding 40% in traditional water treatment processes, while the complexity of their aromatic ring structure further complicates treatment efforts [4]. The enduring nature, bio-toxicity, and degradation difficulty of these organic pollutants present major challenges for water environment management [2,5]. This has driven researchers to seek interdisciplinary solutions aimed at efficient pollutant elimination and mitigation of human health risks.

In current dyeing and printing wastewater treatment methodologies, Advanced Oxidation Processes (AOPs) have emerged as a significant means in the domain of water treatment. This is largely due to their capacity to produce hydroxyl radicals (·OH), which possess high oxidation potential and robust oxidation capability, enabling rapid degradation of organic pollutants while maintaining a straightforward process operation [6]. Among these technologies, photocatalysis has garnered considerable attention owing to its potential for solar-driven oxidation processes [7]. It is viewed as a green and sustainable dye degradation technology, showing substantial promise in realizing the sustainable principles of water treatment.

In recent years, metal sulfide nanomaterials have attracted much attention as photocatalysts because of their simple preparation, excellent electrical and optical properties, and suitable bandgap potential [8]. For example, researchers have studied the photocatalyst performance of metal sulfides such as Cadmium sulfide (CdS) [9,10] and Copper sulfide (CuS) [11]. Copper sulfide, a narrow-bandgap semiconductor material (band gap ~1.7–2.0 eV), demonstrates superior light absorption performance in the visible light range and forms photogenerated electron–hole pairs, thereby establishing its activity in photocatalytic reactions [12]. At present, CuS hollow nanostructures, characterized by their low density and high specific surface area, exhibit potential applications in fields such as medicine, materials, and chemical engineering [13,14,15]. Studies have shown that the smaller-diameter CuS nanoparticles exhibit superior photocatalytic performance [16]. This suggests that within a certain particle size range, the size of CuS particles is directly related to the intensity of light absorption and photocatalytic degradation efficiency. Consequently, regulating the particle size distribution of CuS nanoparticles to expose more active sites for achieving high photocatalytic performance has become a focus of much attention. However, CuS nanoparticles are susceptible to agglomeration during high-temperature sintering, resulting in inadequate exposure of active sites and significantly limiting their catalytic activity [17]. Furthermore, the use of single CuS as a catalyst presents an issue, namely, the rapid recombination of photogenerated electron–hole pairs, which further constrains the enhancement of catalytic efficiency [18]. Consequently, there is an increasing focus in this field on exploring material design strategies that promote efficient dispersion and stability of CuS while simultaneously boosting light absorption and carrier separation efficiency. The journal “*Science*” identified aerogels as one of the top ten emerging science and technology topics in 2021, referring to them as “a transformative new material with significant potential” [19]. S. Zhai et al. suggest that when carbon materials are combined with CuS nanoparticles, they create a synergistic catalytic system. This system markedly improves the photogenerated charge separation efficiency and effectively addresses the inherent limitations of CuS nanoparticles [20]. Notably, carbon aerogels derived from biomass offer distinct advantages. Not only are these aerogels cost-effective and sustainable, but their plentiful oxygen-containing functional groups can anchor metal sulfide nanoparticles, thereby preventing aggregation and facilitating interfacial charge transfer [21,22]. In particular, composite carbon aerogel nanofibers exhibit properties like exceptional biocompatibility, degradability, high conductivity, extensive specific surface area, and impressive carbon retention rate. Their pronounced conductivity expedites the movement of photogenerated electrons, effectively inhibits the recombination of electron–hole pairs, and augments charge separation efficiency. Furthermore, the adsorptive capabilities of carbon aerogel materials can concentrate pollutants on the catalyst surface through an “adsorption–degradation” synergistic mechanism, reducing the mass transfer distance and consequently enhancing the photocatalytic kinetic efficiency [23]. This presents a novel method for addressing the intrinsic limitations of CuS catalysts. Furthermore, the utilization of biomass cellulose carbon aerogel materials can decrease energy consumption and carbon emissions, a factor of significant importance for environmental conservation and sustainable development.

This study synthesizes a CuS/CA composite catalyst using the sol–gel method, which is simple, cost-effective, and efficient. The resultant composite catalyst effectively absorbs visible light. The agglomeration issue of CuS nanoparticles is addressed by anchoring CuS on cellulose aerogel. Furthermore, the carbon aerogel carrier enhances the adsorption rate of dye molecules and accelerates the transmission of photogenerated electrons due to its light porosity, huge specific surface area and thermal stability of carbon aerogel carrier. This helps inhibit the recombination of electron–hole pairs, thereby improving charge separation efficiency and achieving rapid and efficient degradation of organic dyes. The aim of this study is to anchor CuS nanoparticles on the surface of cotton short staple carbon aerogel to create a new type of CuS/CA composite material, and to conduct an in-depth analysis of the synergistic mechanism of adsorption–photocatalysis in this system. Given its simple synthesis method and the high-value utilization of biomass materials, the CuS/CA composite material has broad application prospects as a potential candidate for the efficient treatment of industrial wastewater.

## 2. Results and Discussion

### 2.1. Sample Characterization

#### 2.1.1. XRD Analysis

**Figure 1:**
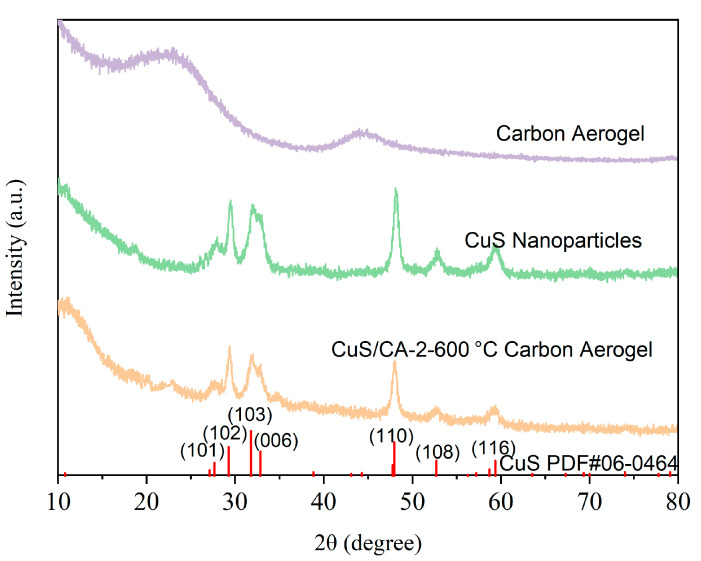
XRD of CuS and Composite Carbon Aerogels.

Based on the XRD pattern presented in Figure 1, the distinct diffraction peaks observed at 2θ = 27.68°, 29.27°, 31.78°, 47.94°, and 59.34° are indexed to the (101), (102), (103), (110), and (116) crystal planes of CuS, respectively. The sharp nature of these peaks indicates the high crystallinity of the synthesized CuS. Furthermore, the peak positions align well with the standard JCPDS reference card (No. 06-0464), confirming the successful formation of a well-crystallized CuS phase.

The XRD pattern of carbon aerogel exhibits a broad peak between 20 and 25°, corresponding to amorphous carbon. Following calcination of the CuS/CA composite at 600 °C under nitrogen atmosphere, characteristic CuS peaks appear in the composite, confirming the effective incorporation of CuS. Following calcination of the composite material at 600 °C under nitrogen atmosphere, the resulting carbon aerogel exhibits an amorphous or highly disordered arrangement. This prevents the formation of sharp peaks in the XRD spectrum, confirming the presence of carbon in the composite as amorphous carbon.

#### 2.1.2. Structural and Compositional Analysis

**Figure 2:**
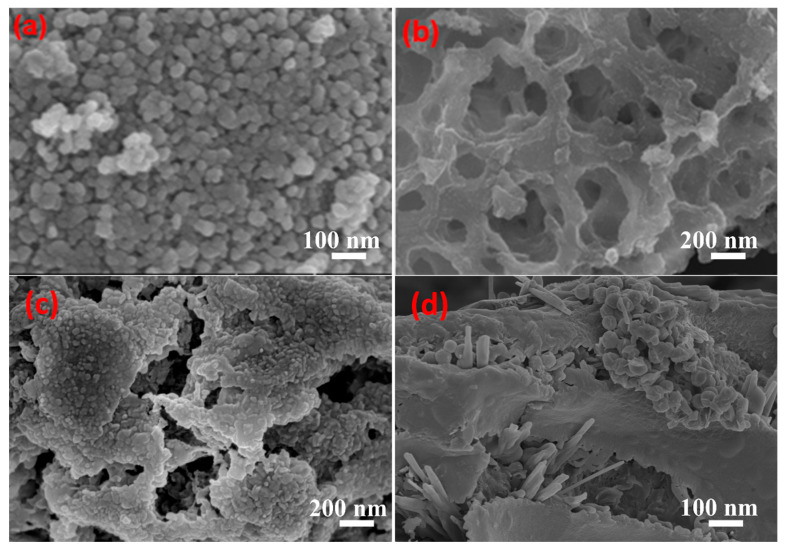
SEM images: (**a**) CuS, (**b**) Cellulose aerogel, (**c**) CuS/CA-2 aerogel, (**d**) CuS/CA-2-600 °C carbon aerogel.

**Figure 3:**
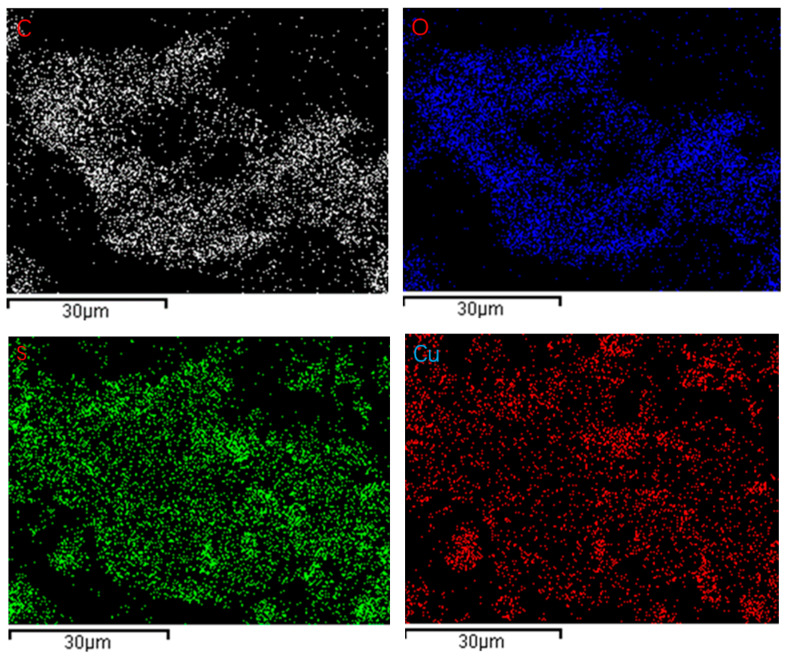
EDS elemental mapping showing the spatial distribution of C, O, S and Cu in the CuS/CA-2-600 °C aerogel.

The Scanning Electron Microscope (SEM) is utilized to investigate the morphology and microstructure of materials. Figure 2a showcases the SEM image of CuS nanoparticles. From the image, it can be observed that the CuS nanoparticles possess a tendency towards regularity in shape, presenting as nearly spherical and similar in size. This indicates that the fabrication process of the material has been well-controlled, which is beneficial for the separation of electrons and holes, as well as the stability of catalysts, ultimately facilitating the photocatalytic degradation of organic pollutants. Figure 2b reveals the internal structure of cellulose carbon aerogels, exhibiting an extremely loose and porous architecture. This characteristic structure offers high mechanical strength and low thermal conductivity, rendering it optimal for the loading of other substances [24,25]. In Figure 2c, CuS nanoparticles can be observed to be loaded onto the surface of the fibrous cellulose aerogel, forming a compact composite material. As depicted in Figure 2d, even after undergoing carbonization treatment, CuS nanoparticles can still be loaded onto the surface of a net-like substrate, which is highly likely composed of a cellulose aerogel framework consisting of C-C bonds. The retention of the continuous macroporous structure in cellulose-derived aerogels after calcination at 600 °C under N_2_ is evidenced by the preserved network architecture, which facilitates uniform dispersion of CuS nanoparticles within the carbon matrix. Elemental mapping (Figure 3) confirms spatial co-localization of C, O, S, and Cu throughout the CuS/CA-2-600 °C carbon aerogel, substantiating the formation of a homogeneous composite with intact nanoscale integration.

#### 2.1.3. XPS

**Figure 4:**
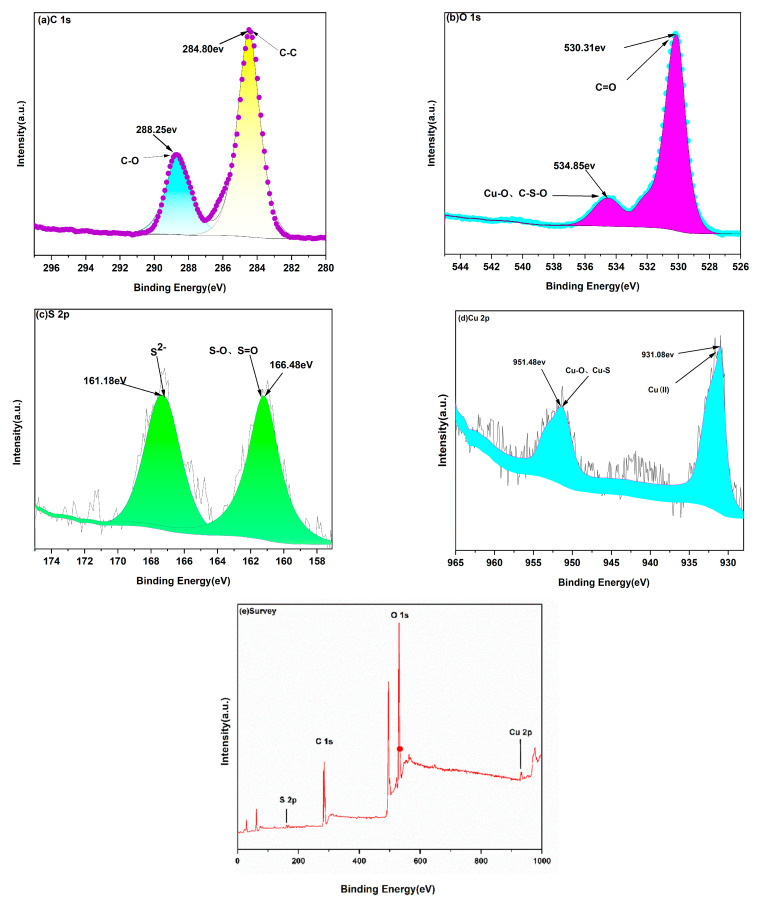
High-resolution XPS spectra of the CuS/CA-2-600 °C carbon aerogel: (**a**) C 1s, (**b**) O 1s, (**c**) S 2p, and (**d**) Cu 2p, along with (**e**) the survey spectrum.

X-ray photoelectron spectroscopy (XPS) analysis of the composite (Figure 4e) evidences C, O, S, and Cu on the surface, with charge referencing established via the C1s peak at 284.8 eV. The high-resolution fine spectrum of C1s in the spectrum of CuS/CA-2-600 (Figure 4a) exhibits peaks at binding energies of 284.80 eV and 288.00 eV, which may correspond to C-C and C-O bonds. This suggests that at this temperature, the cellulose-based carbon aerogel scaffold still retains C-C bonds on the surface of CuS nanoparticles. In the O 1s XPS spectrum (Figure 4b), two distinct peaks are observed at 530.18 eV and 534.48 eV. The lower-binding-energy component (530.18 eV) is assigned to lattice oxygen in Cu-O and C-S-O moieties, likely originating from surface oxidation of CuS during synthesis or exposure to air. Although CuS is primarily composed of Cu and S, trace oxygen is commonly incorporated into the surface layer, forming metal-oxygen or sulfur-oxygen linkages. The higher-binding-energy peak at 534.48 eV is attributed to oxygen in organic functional groups—such as C=O or C-O—possibly derived from residual carbonaceous species, surfactants, or incomplete removal of precursors during processing. As shown in Figure 4c, the S 2p region exhibits two characteristic signals at 161.18 eV and 166.48 eV, corresponding to different sulfur oxidation states. The peak at 161.18 eV is consistent with S^2−^ in stoichiometric CuS, confirming the presence of sulfide anions in the crystal lattice. In contrast, the higher-binding-energy feature at 166.48 eV is indicative of oxidized sulfur species, such as S-O or S=O bonds, which may arise from surface sulfates (e.g., CuSO_4_ or adsorbed SO_4_^2−^) formed via partial oxidation of CuS under ambient or thermal conditions.

These sulfur compounds with oxidation can form S=O or S-O bonds with oxygen and enrich on the sample surface. These chemical bonds have higher binding energies and typically result in the characteristic peak at 166.48 eV. In Figure 4d, the characteristic peaks of Cu elements at 931.08 eV and 951.48 eV possibly correspond to different chemical states. The binding energy of the Cu 2p_3_/_2_ peak at 931.08 eV, in the absence of significant shake-up satellite features, suggests the dominant presence of Cu(II) in the material. This is because copper typically exhibits multiple oxidation states, such as being oxidized to Cu(II) or reduced to Cu(I) or Cu(0) in chemical reactions. In XPS spectra, the binding energy of Cu(II) in the 2p3/2 electron shell is usually around 930 eV, so the observed peak at 931.08 eV in the sample could correspond to Cu(II). The peak at 951.48 eV corresponds to Cu in the form of oxides on the sample surface. In the CuS/CA-2-600 °C carbon aerogel, surface oxidation of copper may lead to the formation of Cu(I)/Cu(II) species (e.g., Cu_2_O or CuO), which can coexist with CuS and participate in Cu-O and Cu-S bonding environments. The XPS signal at 951.48 eV is thus assigned to Cu 2p_1_/_2_ contributions from these mixed copper-chalcogen/oxygen coordination structures.

#### 2.1.4. BET Analysis

**Figure 5:**
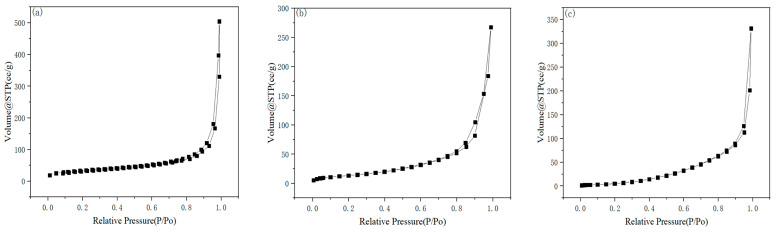
N_2_ Adsorption–Desorption Isotherms and Pore Size Distribution of (**a**) Cellulose Aerogel; (**b**) CuS/CA-2 Aerogel; (**c**) CuS/CA-2-600 °C Carbon Aerogel.

As shown in Figure 5, recording the N_2_ adsorption–desorption isotherms of cellulose aerogel, CuS/CA-2 aerogel, and CuS/CA-2-600 °C carbon aerogel reveals that all three materials conform to the definition of Type IV adsorption isotherms in the IUPAC classification, categorizing them as mesoporous materials. The integration of CuS nanoparticles with a cellulose-derived carbon matrix enhances the effective specific surface area, thereby promoting interfacial contact with organic dye molecules. However, the specific surface area of the resulting composite is highly dependent on the CuS loading level. Excessive CuS incorporation leads to nanoparticle aggregation, which blocks pores and reduces surface accessibility, ultimately diminishing the BET surface area. By comparing Figure 5a–c, it can be noticed that the specific surface area of the composite material also increases to some extent after calcination in a N_2_ atmosphere (as shown in Table 1). Thermal decomposition during calcination induces intra-pore wall roughening via volatile species egress, generating additional surface area within the scaffold architecture [26,27]. Concurrently, removal of physisorbed moisture and residual organics eliminates non-specific surface coverage, further enhancing accessible porosity.

#### 2.1.5. EIS Analysis

**Figure 6:**
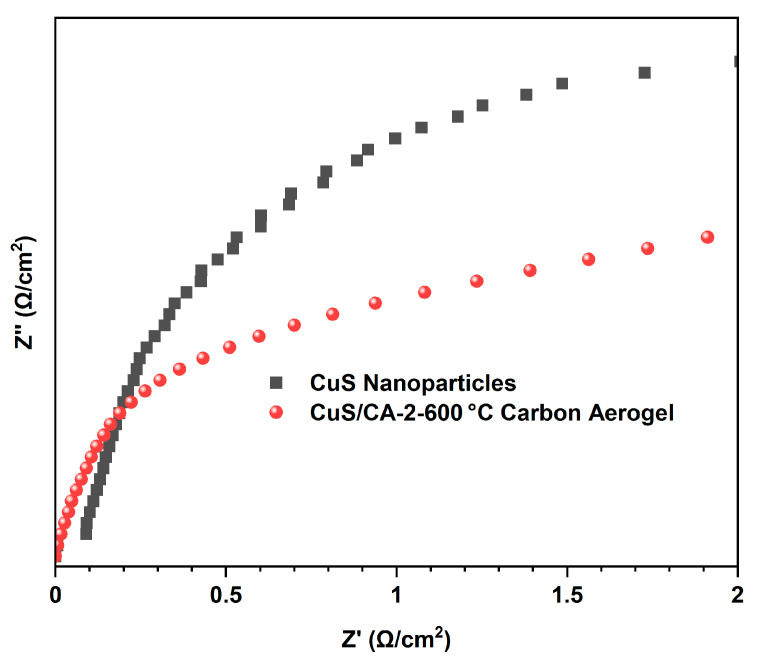
Electrochemical Impedance Spectroscopy.

Electrochemical impedance spectroscopy (EIS) is one of the key techniques to characterize the performance of photocatalytic materials. In the process of photocatalytic degradation, the efficiency of charge separation of photogenerated electrons and holes directly affects the photocatalytic activity of the material. EIS analysis reveals a significantly reduced charge-transfer resistance (R_ct_) in the CuS/CA-2-600 °C carbon aerogel versus CuS nanoparticles, evidenced by the smaller semicircular diameter in the Nyquist plot (Figure 6), indicating enhanced interfacial charge separation critical for photocatalytic efficiency. Specifically, the impedance spectrum radius of CuS nanoparticles is markedly larger than that of CuS/CA-2-600 °C carbon aerogel. This indicates that the formation of the carbon matrix promotes the separation of photogenerated electron–hole pairs, thereby enhancing the overall photocatalytic activity of the composite material.

#### 2.1.6. UV-Vis Diffuse Reflectance Spectroscopy Analysis

**Figure 7:**
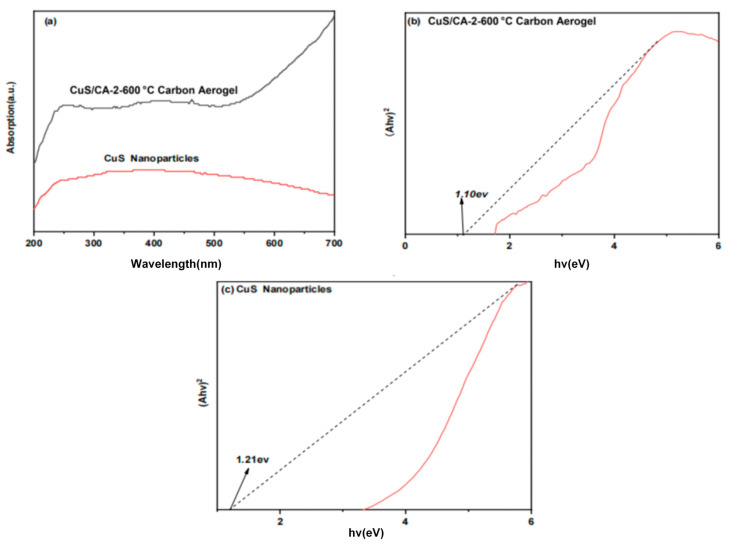
(**a**) UV-Vis diffuse reflectance spectra; Tauc plot graphs of (**b**) CuS nanoparticles; (**c**) CuS/CA-2-600 °C carbon aerogel.

UV-Vis diffuse reflectance spectroscopy quantified optical absorption edges and bandgap energies for photocatalytic assessment. As shown in Figure 7a, this technique was used to test CuS and CuS/CA-2-600 °C carbon aerogel. These materials all exhibit absorption of visible light, with CuS/CA-2-600 °C carbon aerogel showing superior absorption performance throughout the entire range of wavelengths. Therefore, compared to CuS and CuS/CA-2-600 °C carbon aerogel exhibits enhanced visible-light harvesting efficiency, indicating its strong potential as a visible-light-responsive photocatalytic material. The remarkable optical properties of the CuS/CA-2-600 °C carbon aerogel originate from the distinct yet complementary characteristics of cellulose and CuS. The cellulose-derived carbon framework offers a renewable and biocompatible matrix, while the incorporated CuS semiconductor enables visible-light absorption and energy conversion. This strategic integration yields a composite material with improved absorption characteristics and inherent sustainability. Bandgap analysis via the Tauc method (Figure 7b,c) reveals that the CuS/CA-2-600 °C carbon aerogel possesses a narrowed band gap of 1.10 eV, notably smaller than that of pure CuS (1.21 eV), thereby favoring enhanced photoexcitation and charge generation. These factors collectively dictate the optical absorption capacity and band gap of the material, which directly influence its photocatalytic activity for pollutant degradation [28]. Therefore, the composite material formed by CuS and carbon aerogel can generate a narrow band gap. The lower the energy required to excite photons in a narrow band gap, the longer the corresponding absorption wavelength. This provides an effective strategy for further enhancing the photodegradation efficiency of organic pollutants.

### 2.2. Mechanistic Insights into the Adsorption and Photocatalytic Degradation of MB over CuS/CA Carbon Aerogels

#### 2.2.1. Performance Testing

**Figure 8:**
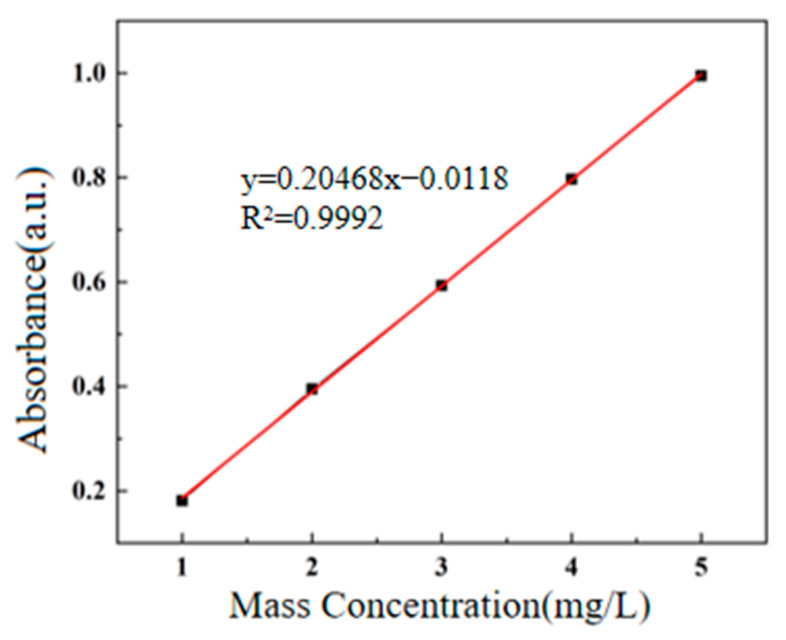
Standard Curve of Methylene Blue Solution.

Photocatalytic activity was evaluated using methylene blue as a representative organic pollutant under simulated solar irradiation provided by a xenon arc lamp. To maintain uniform dispersion and maximize solid–liquid interfacial contact, the reaction suspension was magnetically stirred continuously during irradiation. Prior to irradiation, the system was kept in the dark for 2 h to allow adsorption–desorption equilibrium to be established, thereby enabling clear differentiation between adsorptive uptake and genuine photocatalytic degradation. The concentration of MB was monitored by UV-Vis spectrophotometry, with the characteristic absorption peak identified at 664 nm through full-spectrum scanning. As shown in Figure 8, a calibration curve was constructed from standard solutions of known MB concentrations, yielding a linear regression of *A* = 0.20468*C* − 0.0118 (*R*^2^ > 0.99), where A is absorbance and C is concentration (mg·L^−1^). During illumination, aliquots were withdrawn at predetermined time intervals, immediately centrifuged to remove catalyst residues, and analyzed for residual MB concentration based on the established calibration. To mitigate potential thermal artifacts arising from prolonged lamp exposure, a water-cooling jacket was integrated around the reactor to maintain a constant ambient temperature (~25 °C). This setup ensured that observed degradation was primarily attributable to photocatalytic processes rather than photothermal effects.

#### 2.2.2. The Impact of Workload

**Figure 9:**
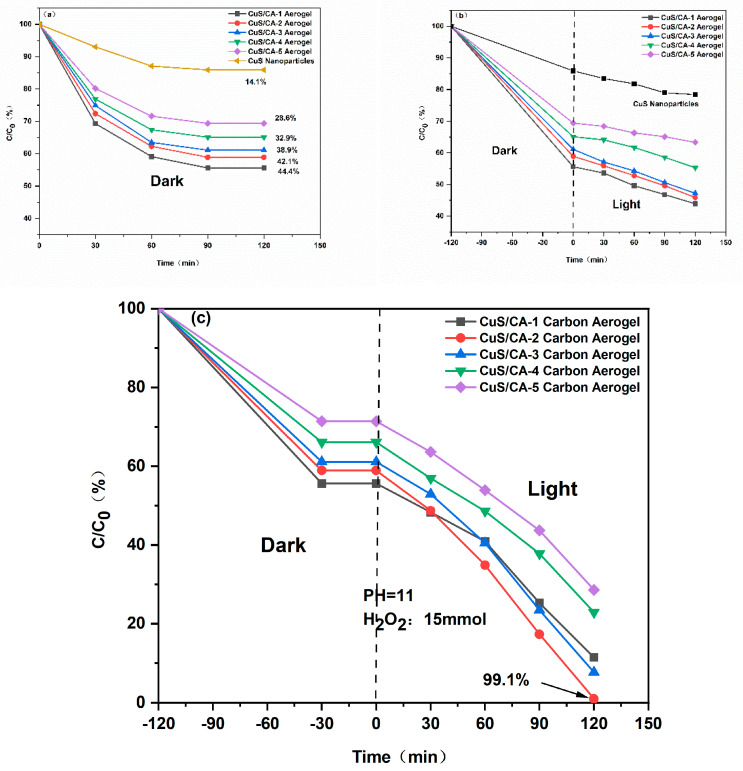
(**a**) Adsorption equilibrium of MB at varying catalyst loadings. (**b**) Photocatalytic degradation profiles of MB under identical irradiation conditions with different catalyst loadings. (**c**) Combined adsorption–photodegradation behavior after optimization of the reaction environment.

To evaluate the adsorption and photocatalytic degradation efficiency of cellulose aerogel-copper sulfide composites, systematic experiments were conducted with varying loading ratios of the active component. As illustrated in Figure 9, the CuS/CA-2-600 °C carbon aerogel displayed markedly enhanced removal performance toward methylene blue relative to other compared materials. These results verify that rational regulation of the cellulose-to-CuS ratio constitutes an effective strategy for optimizing the composite’s adsorption and degradation functions. With increasing CuS loading, the cellulose matrix becomes saturated, and excess CuS nanoparticles tend to agglomerate due to insufficient anchoring sites and enhanced interparticle interactions, thereby diminishing its interaction with cellulose and consequently reducing the adsorption performance towards methylene blue. Eventually, this reaches a limit. This finding indicates the importance of designing the loading ratio appropriately in the preparation of composite materials to achieve the best catalytic effect. Furthermore, by comparing the photocatalytic degradation results shown in Figure 9b,c, it is evident that under non-optimized reaction conditions, even with the addition of CuS nanoparticles, the photocatalytic degradation efficiency of CuS/CA-2-600 °C carbon aerogel towards methylene blue remains insignificant. Therefore, to maximize the photocatalytic efficiency of the composite material in practical applications, precise regulation of key degradation environmental parameters—including pH value and dissolved oxygen concentration—is paramount. In Figure 9c, the CuS/CA-2-600 °C carbon aerogel exhibits the fastest degradation rate between 0 and 30 min, primarily attributable to its optimal CuS loading. CuS functions as the active photocatalytic component. CuS functions as the active component for photocatalysis. An excessively low loading implies a limited number of photocatalytically active sites available for reaction, resulting in a slow degradation rate and consequently impaired performance. Conversely, an excessively high CuS loading readily agglomerates on the carbon aerogel surface to form larger particles, which reduces the exposed surface area of active sites and similarly leads to a decreased degradation rate.

#### 2.2.3. Effect of Carbonization Temperature

**Figure 10:**
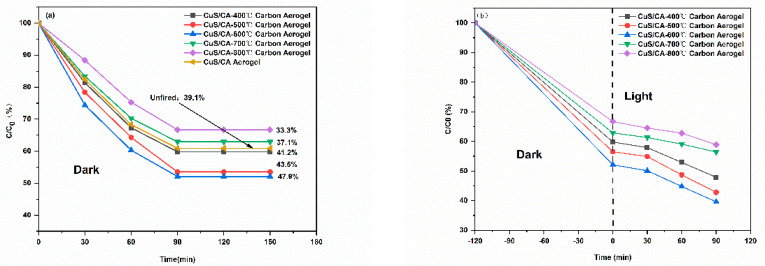
(**a**) Equilibrium adsorption and (**b**) photocatalytic degradation profiles of the composites prepared at different carbonization temperatures.

The adsorption and photocatalytic degradation of MB were investigated over CuS/CA-2 aerogels subjected to carbonization at varying temperatures. As shown in Figure 10a, the sample pyrolyzed at 600 °C exhibits the highest adsorption capacity. This enhancement is ascribed to the thermal activation process, wherein elevated temperatures drive the outward diffusion of volatile species and induce the formation of well-defined pore walls, leading to a marked increase in specific surface area. Concurrently, calcination effectively eliminates physisorbed water and residual impurities, further promoting pore accessibility and surface cleanliness. Beyond this optimal temperature, however, adsorption performance deteriorates—most plausibly due to thermal degradation of the aerogel framework, manifested as pore collapse or coalescence under excessive pyrolysis conditions.

#### 2.2.4. The Impact of pH and H_2_O_2_

**Figure 11:**
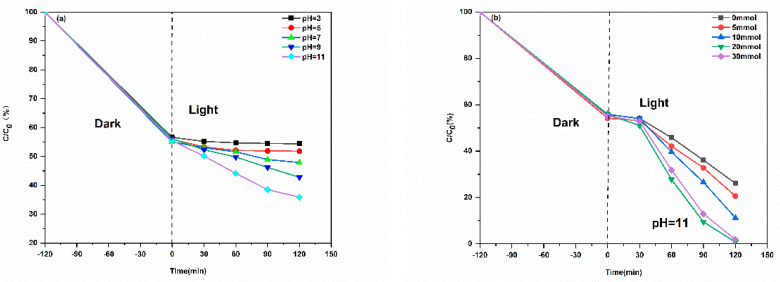
(**a**) Adsorption equilibrium of methylene blue (MB) over CuS/CA−2 aerogels carbonized at different temperatures; (**b**) Corresponding photocatalytic degradation profiles under identical irradiation conditions.

To elucidate the influence of reaction medium on the photocatalytic activity of the CuS/CA-2-600 °C carbon aerogel, systematic degradation experiments were conducted under varying pH values and H_2_O_2_ dosages. As shown in Figure 11a, the MB removal efficiency is markedly pH-dependent. Under acidic conditions (e.g., pH < 5), degradation performance is severely limited—likely due to the acid-induced dissolution of CuS, which compromises catalyst stability and active site availability. In contrast, alkaline conditions (particularly pH = 11) dramatically enhance MB degradation, achieving a removal rate of 69% within just 120 min for a 20 mg·L^−1^ MB solution.

This improvement can be rationalized by the favorable alkaline environment for H_2_O_2_ activation. Under high pH, H_2_O_2_ readily decomposes to generate hydroxyl radicals (•OH), which subsequently react with sulfide species (S^2−^) on the CuS surface to produce sulfur-centered radicals. These reactive intermediates synergistically accelerate dye oxidation, thereby boosting overall photocatalytic efficiency. Consequently, an alkaline milieu proves optimal for the CuS/H_2_O_2_ photocatalytic system. The dosage of H_2_O_2_ also critically modulates degradation kinetics (Figure 11b). An addition of 20 mmol H_2_O_2_ yields the highest MB degradation rate, whereas further increasing the dose to 30 mmol leads to no appreciable improvement—and may even cause slight inhibition. This concentration-dependent behavior aligns with the well-established dual function of H_2_O_2_ in advanced oxidation processes: while it serves as a productive source of hydroxyl radicals (•OH) at optimal concentrations, excessive H_2_O_2_ conversely scavenges reactive oxidative species through competitive reactions, ultimately reducing the overall degradation efficiency. Thus, the optimal photocatalytic performance is achieved under the combined conditions of pH 11 and 20 mmol H_2_O_2_ addition, highlighting the importance of fine-tuning both chemical and operational parameters to maximize the efficacy of sulfide-based photocatalytic systems.

#### 2.2.5. Exploring the Reusability Performance of Catalysts

**Figure 12:**
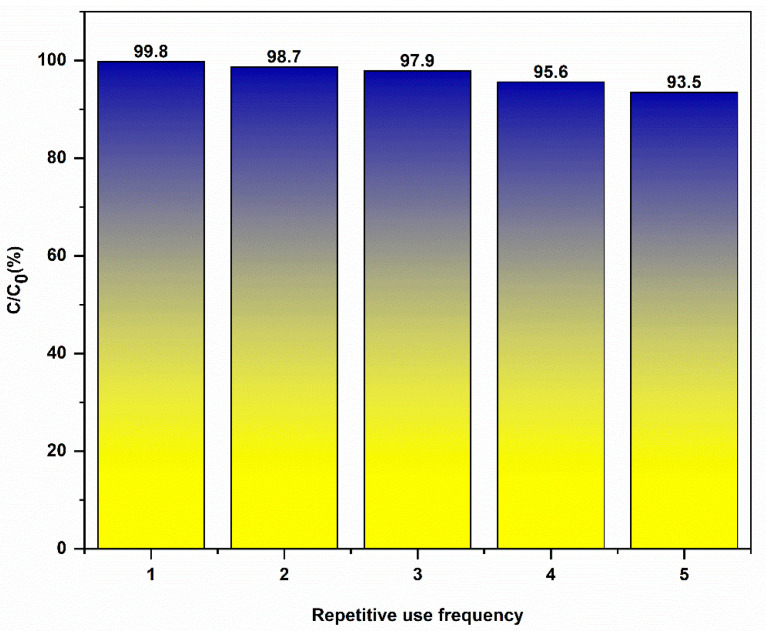
Repeatability of Catalyst.

After five cycles of experimentation (Figure 12), it was observed that CuS/CA-2-600 °C carbon aerogel exhibits an impressive degradation efficiency towards methylene blue. The composite material demonstrates robust cycling stability, as evidenced by its maintained removal rate exceeding 93.5% throughout multiple adsorption–photodegradation cycles. Although a gradual decline in efficiency is observed with increasing cycle number, this attenuation is mainly ascribed to the residual adsorption of MB molecules at the photocatalyst surface, which reduces the available active sites under repeated use. Nevertheless, the material retains high reliability and reproducibility, underscoring its potential for sustained practical application in pollutant removal. Hence, the conclusion is that CuS/CA-2-600 °C carbon aerogel is able to maintain its stability throughout the methylene blue degradation process and has a high level of reusability.

Table 2 demonstrates that, compared with catalysts used for methylene blue degradation in the literature, CuS/CA-2-600 °C carbon aerogel exhibits outstanding cyclical stability: after multiple cycles, its activity exhibits minimal decay whereas similar catalysts generally show significant degradation. This indicates that CuS/CA-2-600 °C carbon aerogel holds considerable promise for practical applications.

#### 2.2.6. Radical Trapping Experiments

**Figure 13:**
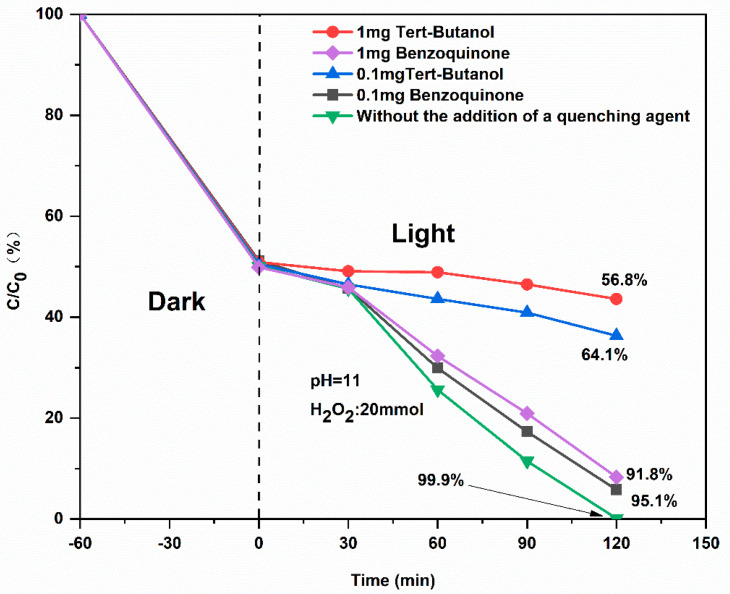
The alterations in methylene blue concentration under various control groups.

Radical trapping experiments were conducted to identify the dominant reactive species involved in the photocatalytic degradation of MB over the CuS/CA carbon aerogel. As shown in Figure 13, in the absence of any scavenger, near-complete MB removal (>99.9%) is achieved within 120 min. In stark contrast, the introduction of tert-butanol (•OH scavenger) drastically suppresses degradation: with 1 mg of tert-butanol, the MB removal efficiency drops to below 12% after 120 min, indicating that the catalytic process is almost entirely inhibited. Even at a lower dosage (0.1 mg), degradation efficiency remains below 20%, underscoring the critical dependence of the system on hydroxyl radicals. Conversely, the addition of benzoquinone (a superoxide radical, •O_2_^−^, scavenger) results in only a marginal decrease in degradation rate, suggesting that •O_2_^−^ plays a secondary or auxiliary role in the reaction pathway. Collectively, these results unambiguously identify hydroxyl radicals (•OH) as the dominant reactive species responsible for MB degradation in the CuS/CA-based Fenton-like system, with superoxide radicals (•O_2_^−^) playing only a minor role in the overall oxidation pathway [35].

#### 2.2.7. Photodegradation and Mechanism Analysis

**Figure 14:**
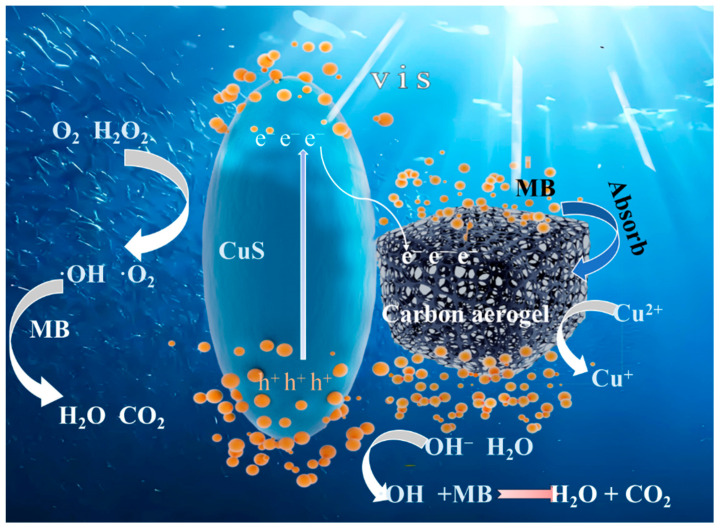
Adsorption-Decomposition Mechanism Diagram.

As shown in Figure 14, the catalytic mechanism in CuS primarily involves adsorption on the surfaces of carbon aerogel and CuS nanoparticles. Upon absorption of energy, the electrons (e^−^) are excited from the valence band (VB) to the conduction band (CB). This leads to oxidation and reduction reactions on the surface of CuS nanoparticles, generating hydroxyl radicals (•OH) and superoxide radicals (•O_2_^−^) as oxidizing agents. The proton (H^+^) provided by the oxidizing agent can facilitate water ionization in organic dye solutions, generating hydroxide ions (OH^−^). In the presence of other strong oxidizing radicals, hydroxyl radicals (•OH) are formed through induced reactions. These decomposed radicals and active oxidizing agents can participate in catalytic reactions, decomposing organic dyes [35]. The addition of H_2_O_2_ can efficiently trap photogenerated electrons, separate them from holes, and thereby increase photon yield and reaction rate. Furthermore, the inclusion of hydrogen peroxide can generate hydroxyl radicals with strong oxidizing properties.

1. H_2_O_2_ + e_cb_^−^ → •OH + OH^−^
2. H_2_O_2_ + O_2_ → •OH + OH^−^ + •O_2_^−^
3. O_2_ + e_cb_^−^ → •O_2_^−^
4. Cu^2+^ + H_2_O_2_ → H^+^ + CuOOH^+^
5. CuOOH^+^ → HOO^−^ + Cu^+^
6. Cu^+^ + H_2_O_2_ → Cu^2+^ + •OH + OH^−^

The •OH hydroxyl radicals, OH^−^ hydroxide ions, e_cb_^−^ electron holes, and •O_2_^−^ superoxide radicals are present. It is stated that the intermediate formed from the reaction between Cu^2+^ ions and H_2_O_2_ can create a system similar to a Fenton catalyst, generating hydroxyl radicals. These hydroxyl radicals exhibit strong oxidizing properties. The Cu^2+^/Cu^+^ cycle promotes the production of •OH radicals, which act on the adsorbed MB on cellulose aerogel and degrade it into smaller molecules. Through further transformation, CO_2_ and H_2_O are formed.

When used alone, CuS suffers from limited photocatalytic efficiency—primarily due to its modest quantum yield—which restricts its viability in practical environmental remediation. However, the catalytic performance can be markedly enhanced by incorporating a small amount of hydrogen peroxide (H_2_O_2_) into the reaction system, thereby establishing a Cu^2+^/H_2_O_2_-based Fenton-like process. In this configuration, Cu^2+^ ions derived from CuS serve as redox-active centers that catalyze the decomposition of H_2_O_2_, generating highly reactive oxygen species such as hydroxyl radicals (•OH) and, to a lesser extent, superoxide radicals. These radicals drive rapid oxidative degradation of organic pollutants. The synergy between the carbon-supported CuS and H_2_O_2_ thus transforms the composite into a potent heterogeneous Fenton-like catalyst, significantly accelerating contaminant mineralization under mild conditions.

#### 2.2.8. Photocatalytic Treatment of Simulated Reactive Dyeing Wastewater

Simulated printing and dyeing wastewater containing Reactive Red 24 (RR24) and Reactive Yellow 145 (RY145) was prepared according to the industrial formulation ratios listed in Table 3 and Table 4, which reflect standard process recipes for self-dispersible K-type and M-type reactive dyes. The concentrations of RR24 and RY145 were determined based on their respective dyeing protocols, ensuring accurate representation of typical dye loadings in real effluents. In addition to the dyes, auxiliary chemicals commonly employed in industrial dyeing—such as electrolytes, alkali agents, and dispersants—were incorporated at concentrations specified in the process recipes. All components were precisely dosed to maintain strict adherence to the prescribed formulations, thereby replicating the chemical complexity of actual textile wastewater. This approach ensures that the experimental conditions closely mimic real-world scenarios, enhancing the reliability and applicability of the photocatalytic degradation results [36,37].

(1) Photocatalytic degradation was evaluated using a simulated printing and dyeing wastewater spiked with Reactive Red 24 and Reactive Yellow K as representative pollutants. A fixed dosage of 10 mg of the CuS/cellulose-derived carbon aerogel was introduced into the system, and a xenon lamp was used to simulate solar irradiation. To ensure homogeneous dispersion and intimate contact between the catalyst and the dye solution, the reaction mixture was continuously agitated with a magnetic stirrer throughout the experiment. A water-cooling jacket was integrated around the reactor to dissipate heat generated during prolonged illumination, thereby maintaining a stable reaction temperature and minimizing thermal artifacts. A 1 h dark adsorption period was implemented before irradiation to establish adsorption–desorption equilibrium, enabling clear distinction between adsorptive removal and genuine photocatalytic degradation. Aliquots were withdrawn at predetermined time intervals, and the residual dye concentration was quantified by monitoring the characteristic absorbance of the solution via UV-Vis spectrophotometry.

(2) Industrial reactive dyeing processes commonly employ inorganic electrolytes—primarily NaCl or Na_2_SO_4_—to enhance dye exhaustion and fixation, yielding effluents with elevated Na^+^ concentrations. This high ionic strength environment impairs photocatalytic degradation by inducing aggregation of anionic dye molecules, thereby reducing their availability at catalytic interfaces. The destabilizing effect of Na^+^ on dye dispersion stems from two interrelated phenomena, the first being hydrolysis suppression and the salting-out effect. Reactive dyes typically bear sulfonate (–SO_3_^−^) or carboxylate (–COO^−^) groups, rendering them negatively charged in aqueous solution. In low-salt conditions, electrostatic repulsion between like-charged dye anions ensures colloidal stability. However, as Na^+^ concentration increases, the electrical double layer surrounding dye molecules is compressed, screening surface charges and suppressing the dissociation of dye–Na^+^ ion pairs. This reduces the population of free dye anions and weakens the electrostatic barrier against aggregation. Consequently, van der Waals forces dominate, driving molecular association and potentially leading to precipitation. Such aggregation not only alters the physical state of the dye but also limits its diffusion to and interaction with photocatalytic active sites, ultimately diminishing degradation efficiency.

**Figure 15:**
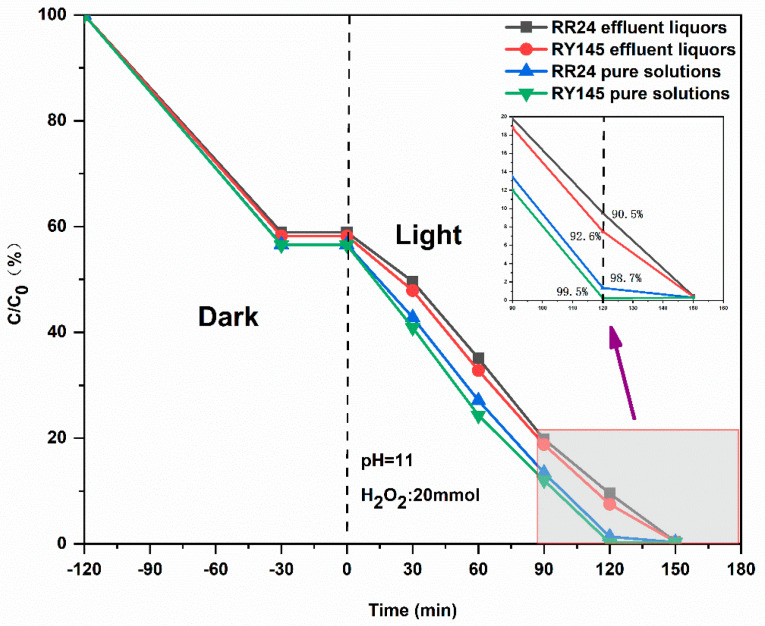
Photocatalytic degradation of dyes in complex wastewater matrices.

Figure 15 compares the UV-driven photocatalytic performance of the CuS/CA composite carbon aerogel toward Reactive Red 24 and Reactive Yellow 145 in both pure aqueous solutions and simulated printing and dyeing wastewater. Despite the presence of inorganic sodium salts—which are known to suppress dye degradation through aggregation and charge screening—the inhibitory effect remains moderate. Notably, extending the irradiation time compensates for this suppression, enabling degradation efficiencies comparable to those achieved in pure dye solutions. For Reactive Red 24, after 120 min of ultraviolet light irradiation, its degradation rate in pure solution reached 98.7%, while in dyeing and printing wastewater, the degradation rate was 90.5%. After 150 min of irradiation, the photocatalytic system achieved near-complete degradation of Reactive Red 24 in the simulated dyeing wastewater, with a removal efficiency exceeding 99.9%. Similarly, for Reactive Yellow 145, after 120 min of ultraviolet light irradiation, its degradation rate in pure solution was 99.5%, nearly complete degradation, while in dyeing and printing wastewater, the degradation rate was 92.6%. Extending the irradiation time to 150 min resulted in near-complete degradation of Reactive Yellow 145 in the wastewater. Nevertheless, the presence of high concentrations of Na^+^ ions in the effluent slightly suppressed the UV-driven photocatalytic degradation efficiency of reactive dyes, likely due to charge screening and reduced electrostatic repulsion among dye molecules, as discussed previously. However, through an extension of time, the catalyst can still achieve nearly complete degradation of reactive dyes within 150 min.

## 3. Conclusions and Outlook

Using cotton linter cellulose as a sustainable precursor, a series of CuS/cellulose derivative aerogel (CuS/CA) composites with tunable copper sulfide loading were successfully prepared via a combined hydrothermal–freeze-drying–carbonization strategy. The porous structure of the carbon aerogel effectively suppressed the agglomeration of ultrafine CuS nanoparticles while significantly enhancing adsorption capacity and photocatalytic activity towards organic pollutants. Through the catalytic degradation of methylene blue solution, a CuS loading of 20% (mass fraction) and a carbonization temperature of 600 °C were determined as optimal conditions, with pH 11 and H_2_O_2_ concentration of 20 mmol providing the most favorable degradation environment. Furthermore, the optimized CuS/CA-2-600 °C carbon aerogel was subsequently employed for the catalytic degradation of simulated textile wastewater containing K-type and M-type reactive dyes. Under illumination, this catalyst achieved degradation efficiencies of 90.5% for Reactive Red 24 and 92.6% for Reactive Yellow 145 within 120 min, with near-complete removal (>99.9%) attained after 150 min. This study collectively demonstrates that CuS/CA-2-600 °C carbon aerogel exhibits low cost, high stability, and high efficiency as a photocatalyst for organic pollutant removal. This technology provides a feasible and scalable solution for treating complex dyeing wastewater, pioneering a new pathway for sustainable water treatment by integrating biomass upgrading with advanced oxidation techniques.

Despite achieving certain research progress, several new issues requiring urgent resolution and improvement have emerged during the investigation. Moving forward, we shall delve deeper into the adsorption theory and kinetic models of photocatalysts. Employing theoretical calculations and quantum chemical methods, we will conduct more thorough investigations into the photocatalytic reaction mechanisms of CuS/CA photocatalysts. This will involve exploring the intrinsic relationships between factors such as charge carrier structure, dopant elements, and interfacial interactions, thereby providing theoretical guidance for the optimized design of catalysts.

## 4. Materials and Methods

### 4.1. Materials and Reagents

Cotton linters (cellulose, (C_6_H_10_O_5_)ₙ) were provided by Aksu Short Fiber Co., Ltd. (Aksu, China). Thioacetamide (CH_3_CSNH_2_, ≥99%), copper(II) sulfate pentahydrate (CuSO_4_·5H_2_O, ≥99%), sodium hydroxide (NaOH, pellets, ≥98%), methylene blue (C_16_H_18_ClN_3_S, ≥99%), N,N′-methylenebisacrylamide (C_7_H_10_N_2_O_2_, ≥99%), anhydrous ethanol (C_2_H_6_O, ≥99.5%), and urea (CO(NH_2_)_2_, ≥99%) were all of analytical grade and used as received. Ammonia solution (25% NH_3_ in H_2_O) was purchased from Tianjin Yongsheng Fine Chemical Co., Ltd., Tianjin, China. Deionized water (resistivity ≥ 18.2 MΩ·cm) was used throughout all experiments.

### 4.2. Preparation of CuS/CA Nanocomposites

Cotton short fibers (5.0 g) were subjected to sequential purification and mechanical disintegration in distilled water, followed by thermal drying at 60 °C for 6 h to ensure complete moisture removal. The dried biomass was homogenized in a pre-cooled aqueous mixture of NaOH, urea, and water (7:12:81, mass ratio) to achieve complete dissolution, yielding a homogeneous cellulose solution upon complete dissolution. This solution was cryo-induced into a hydrogel network via static freezing at −20 °C for 12 h. Concurrently, CuS nanomaterials were synthesized hydrothermally. Briefly, CuSO_4_·5H_2_O (5.0 g) and thioacetamide (CH_3_CSNH_2_, 1.5 g) were co-dissolved in deionized water (60 mL) under ambient stirring (1 h). The resulting precursor suspension was transferred to a Teflon-lined stainless-steel autoclave and subjected to hydrothermal treatment at 100 °C for 12 h. The precipitated CuS product was recovered via centrifugation, washed with ethanol/water, and dried under vacuum at 60 °C for 8 h prior to storage. Upon thawing at ambient temperature, the pre-formed cellulose hydrogel was mechanically homogenized using a magnetic stirrer (30 min, 500 rpm) to restore colloidal uniformity and facilitate subsequent composite integration.

The mixture was then subjected to centrifugation at 8000 r/min for 10 min. Subsequently, 0.6 g of cross-linking agent (C_7_H_10_N_2_O_2_) was added to a mixture of 20 mL of cellulose hydrogel and CuS, and stirred for 5 h. After that, it was left to stand overnight to form. The resulting product was washed with neutral water and anhydrous ethanol, freeze-dried for 24 h, and the CuS loading content in the cellulose matrix was controlled by varying the amount of CuS introduced during synthesis. The obtained CuS/cellulose composite material was calcined in a tube furnace under N_2_ atmosphere protection for 2 h to obtain the CuS/CA nano-composite catalyst. A series of CuS/cellulose-derived carbon aerogels with varying CuS loadings (10–50 wt%) were designated as CuS/CA-1 to CuS/CA-5, corresponding to 10%, 20%, 30%, 40%, and 50% CuS content, respectively.

### 4.3. Instruments and Characterization

Instrumentation employed for comprehensive material characterization included a UV-Vis spectrophotometer (UV-6100, Shanghai Elementar Analytical Instrument Co., Ltd., Shanghai, China) for optical absorption analysis, complemented by a second UV-Vis system (UV-2550, Shimadzu, Kyoto, Japan) to ensure spectral reproducibility. Structural and chemical surface properties were probed using X-ray photoelectron spectroscopy (ESCALAB Xi^+^, Thermo Fisher Scientific, Waltham, MA, USA). Crystalline phase identification was performed using X-ray diffraction (D8 Advance, Bruker AXS GmbH, Karlsruhe, Germany) with Cu-Kα radiation. Textural properties, including specific surface area and pore size distribution, were determined via nitrogen physisorption at 77 K using an ASAP 2460 analyzer (Micromeritics Instrument Corporation, Norcross, GA, USA). For photoactivity assessments, a 120 W xenon lamp (Beijing Princeton Technology Co., Ltd., Beijing, China) served as the simulated solar irradiation source, calibrated to match AM 1.5 G conditions where applicable. A high-resolution field emission scanning electron microscope (SU8010, Hitachi, Tokyo, Japan) equipped with an energy dispersive spectrometer (EDS) was employed to analyze the surface morphology, elemental composition, and distribution of the samples.

### 4.4. Performance Testing of CuS/CA Carbon Aerogel Composites

During performance testing, methylene blue and dye wastewater served as pollutants. A 120 W xenon lamp was selected to simulate sunlight for the experiment, with continuous stirring provided by a magnetic stirrer to ensure thorough contact between the composite material and the pollutant solution. To establish the degradation environment, a standard calibration curve for methylene blue was first plotted by measuring the absorbance of solutions at varying concentrations. A linear relationship was observed between mass concentration and absorbance: y = 0.20468x − 0.0118. The full spectrum of the methylene blue solution was scanned using a UV-visible spectrophotometer, yielding a maximum absorption wavelength of 664 nm. To mitigate temperature effects on the experiment, water was continuously introduced for reflux throughout the degradation process. Concurrently, to distinguish degradation from adsorption, the prepared composite material underwent dark treatment. After reaching adsorption equilibrium (120 min), illumination was initiated. Samples were collected at appropriate intervals, with changes in pollutant concentration characterized by variations in the material’s absorbance values. Finally, the composite material was applied to the catalytic degradation of two dyes commonly used in textile dyeing and finishing processes (process formulations for K-type and M-type reactive dyes) to investigate its photocatalytic degradation performance for dye wastewater.

Experimental procedure for assessing catalyst reusability: After washing the spent catalyst with clean water, reintroduce it into a fresh reaction system. Evaluate the catalyst’s reusability by comparing the reaction efficiency across successive experimental cycles.

## Figures and Tables

**Table 1:** Specific surface area of various materials.

Sample	Specific Surface Area (m^2^/g)	Average Pore Size (nm)
CuS	9.53	1.23
Cellulose Aerogel	124.36	11.89
CuS/CA-2 Aerogel	63.21	4.31
CuS/CA-2-600 °C Carbon Aerogel	91.33	3.78

**Table 2:** Comparative Study of the Catalytic Performance of CuS/CA-2-600 °C Carbon Aerogel and Other Catalysts.

Catalyst	Pollutant	Degradation Rate (%)	Degradation Rate After Recycling% (Number of Cycles)	Ref.
MRGO 20	MB	95.2	54.6 (5)	[29]
Fe_3_O_4_@MnO_2_	MB	98	98 (3)	[30]
Fe-glycerate	MB	99.7	61 (4)	[31]
40%Pg-C_3_N_4_/Ag_2_MoO_4_	MB	99.5	85 (4)	[32]
Au/ZnO	MB	90	54.1 (3)	[33]
CdSe/g-C_3_N_4_	MB	89.4	-	[34]
CuS/CA-2-600 °C Carbon Aerogel	MB	99.1	93.5 (5)	This work

**Table 3:** Formulation of simulated RR24 dyeing wastewater.

Title	Concentration	Effects
Dyeing agent 1227	8 mg/L	Even-dyeing agent
NaCl	0.4 g/L	Color accelerator
Rapid penetration agent T	20 mg/L	Penetrating agent
RR24	10 mg/L	Dye
Na_2_CO_3_	0.3 g/L	Fixative
Deionized water	——	Medium

**Table 4:** Formulation of simulated RY145 dyeing wastewater.

Title	Concentration	Effects
Dyeing agent 1227	8 mg/L	Even-dyeing agent
Na_2_SO_4_	0.3 g/L	Color accelerator
Rapid penetration agent T	20 mg/L	Penetrating agent
Na_2_SO_4_	0.3 g/L	Color accelerator
RY145	10 mg/L	Dye
Deionized water	——	Medium

## Data Availability

The original contributions presented in this study are included in the article. Further inquiries can be directed to the corresponding author.
